# Age-stratified clinical characteristics of hand, foot, and mouth disease in children and its guiding value for diagnosis and treatment

**DOI:** 10.3389/fped.2026.1840062

**Published:** 2026-07-07

**Authors:** Shuzhen Dai, Xiaoling Hu, Meihong Lin, Wenwen Chen, Liping Xu

**Affiliations:** Department of Neonatology, Zhangzhou Affiliated Hospital of Fujian Medical University, Zhangzhou, China

**Keywords:** age stratification, cerebrospinal fluid, enterovirus, hand, foot and mouth disease, neurological complications

## Abstract

**Objective:**

To investigate the age-stratified differences in core clinical characteristics of children with hand, foot and mouth disease (HFMD) and their clinical reference value for clinical diagnosis and treatment.

**Methods:**

This single-center retrospective study enrolled 216 hospitalized children with HFMD from January 2022 to December 2024. Patients were stratified into four age groups: ≤1 month, 1 month–1 year, 1–3 years, and >3 years. Epidemiological features, clinical manifestations, laboratory findings, and complications were analyzed using R software.

**Results:**

Children aged 1–3 years accounted for the largest proportion (62.76%), and 88.43% of patients were under 4 years old. Neonates had a higher summer incidence and longer median hospital stay (8 days) than other age groups. Neonates presented with lower rates of rash and herpangina but significantly higher cerebrospinal fluid (CSF) protein and lower CSF glucose levels (*P* < 0.001). The 1 month–1 year group had the highest peripheral white blood cell (WBC) and lymphocyte counts (*P* < 0.001). Convulsions were most frequent in the 1–3 years group (21.48%), while >3 years group had higher rates of intracranial hypertension (24.00%). Neonates had higher rates of meningeal irritation signs (66.67%) and aseptic meningitis (73.33%) (*P* < 0.05). Peripheral WBC and neutrophil counts were positively correlated with C-reactive protein (CRP) and procalcitonin levels; CSF WBC was correlated with CSF protein and peripheral blood leukocyte indices.

**Conclusion:**

HFMD shows significant age-related clinical heterogeneity. Neonatal infections tend to follow a relatively benign course with prominent CSF abnormalities, whereas older children are more prone to neurological complications. Age-stratified diagnostic and management strategies may facilitate early risk identification and improve clinical outcomes.

## 1 Background

Hand, Foot, and Mouth Disease (HFMD), an acute infectious disease in children caused by enteroviruses, remains a common pediatric infectious disease worldwide, particularly in Asia. Its clinical manifestations are highly heterogeneous: mild cases are predominantly characterized by fever, cutaneous exanthema, and oropharyngeal herpes, whereas severe cases can involve the central nervous system, leading to life-threatening complications and even mortality (1–4). Thus, an in-depth exploration of the clinical characteristics of HFMD is of critical significance for the early identification of high-risk pediatric patients and the optimization of clinical diagnosis and treatment strategies.

In terms of epidemiological features, numerous studies have confirmed that HFMD primarily afflicts children under 5 years of age, with distinct seasonal fluctuations and geographical variations in its prevalence (5, 6). For laboratory parameters, several researchers have investigated the associations between white blood cell counts, C-reactive protein (CRP) levels, cerebrospinal fluid (CSF) indices and HFMD disease severity. Most studies have focused on the overall pediatric HFMD population, with limited age-stratified analyses, particularly regarding differences between neonates and older children and the relationship between CSF and peripheral inflammatory markers (7–12). This gap limits the development of age-specific diagnostic and management strategies.

Therefore, the present study aims to conduct a comprehensive and systematic analysis of core clinical, epidemiological and laboratory characteristics in children with HFMD from a rigorous age-stratified perspective. By clarifying the age-specific clinical profiles and intergroup differences of HFMD, we intend to provide evidence-based clinical references for clinicians, thereby facilitating the improvement of clinical management strategies for pediatric HFMD patients across different age groups.

## 2 Materials and methods

### 2.1 Study design

This study was a single-center retrospective cohort study. The research subjects were pediatric patients with hand, foot, and mouth disease (HFMD) admitted to Zhangzhou Hospital Affiliated to Fujian Medical University from January 2022 to December 2024, with study data extracted from the patients' electronic medical records.

Inclusion Criteria: (1) The diagnostic criteria adhered to the Health Industry Standards of the People's Republic of China (WS 588-2018) (13); (2) Age ≤ 14 years; (3) Complete clinical data available, including epidemiological information, records of symptoms and physical signs, laboratory test results, treatment regimens, and prognostic follow-up data.

Exclusion Criteria: (1) Complicated with severe underlying diseases such as congenital heart disease, hereditary metabolic diseases, and primary immunodeficiency diseases; (2) Complicated with bacterial, fungal, or other non-enteroviral infections (e.g., respiratory syncytial virus, adenovirus infections); (3) Missing key clinical information (e.g., undetected core laboratory indicators, insufficient follow-up duration).

A total of 216 eligible pediatric patients were finally enrolled in the study and stratified into four groups according to age. The specific grouping criteria and sample sizes were as follows: ≤1 month group (*n* = 30), 1 month–1 year group (*n* = 26), 1–3 years group (*n* = 135), and >3 years group (*n* = 25).Data collection included the following dimensions: demographic characteristics (gender, age, residential environment), season of disease onset, clinical manifestations (fever duration, maximum body temperature, rash, neurological symptoms, etc.), laboratory indicators [complete blood count, procalcitonin (PCT), C-reactive protein (CRP), cerebrospinal fluid (CSF) tests, and enterovirus genotyping], as well as treatment and outcome information (hospital stay, complications, prognosis). All data were de-identified and used exclusively for the purpose of this study.

### 2.2 Definitions

1. The diagnostic criteria of HFMD adhered to the Health Industry Standards of the People's Republic of China (WS588-2018); Both mild and severe cases were included in this study.
2. Aseptic meningitis was defined as an inflammatory reaction of the meninges, but no evidence of purulent bacterial infection can be found in the CSF through routine bacterial tests.
3. Aseptic encephalitis is defined as brain tissue inflammation caused by non-bacterial pathogens (primarily viruses).
4. Intracranial hypertension was diagnosed based on clinical manifestations (headache, vomiting, altered mental status), increased CSF opening pressure（>20 cmH2O), or radiological evidence.
5. Meningeal irritation signs were judged positive if neck stiffness, Kernig's sign, or Brudzinski's sign was present.
6. Convulsion are defined here as acute symptomatic seizures directly caused by clear acute central nervous system injuries, rather than primary epilepsy.
7. Enterovirus genotyping: Enteroviral nucleic acid in throat swab or CSF samples was detected via reverse transcription-polymerase chain reaction (RT-PCR). Based on primer specificity, the enteroviruses were classified into Enterovirus (EV) 71, CVA16, all other enterovirus strains were classified as “other types”due to methodological restrictions. Further serotypic identification.

### 2.3 Statistical analysis

No prospective sample size calculation was performed, because this was a retrospective observational study. All study data were processed and analyzed using R statistical software (Version 4.3.1). Before data entry, electronic medical record information was independently cross-checked by two researchers to ensure data accuracy; any disputed data were verified by reviewing the original paper medical records.

Missing data were inspected for all key variables including demographic characteristics, clinical manifestations, laboratory results, and complication data. Complete case analysis was adopted; only participants with complete data for the specific analysis were included in each statistical comparison. Given the retrospective nature of the main variables and the relatively low proportion of missing values, data with a missing rate of ≥10% were excluded, and multiple imputation was performed for variables with a missing rate of ＜10%.

Continuous variables with a normal distribution were presented as mean ± standard deviation (Mean ± SD); one-way analysis of variance (one-way ANOVA) was used for intergroup comparisons, and the LSD-t test was applied for *post-hoc* pairwise comparisons. Continuous variables with a non-normal distribution were expressed as median (interquartile range) [M (Q_1_, Q_3_)]; the Kruskal–Wallis H test was used for overall intergroup comparisons, and the Wilcoxon rank-sum test with Bonferroni correction was adopted for *post-hoc* pairwise comparisons.

Categorical variables were presented as frequency (percentage) [n (%)], and the Pearson's chi-square (*χ*^2^) test was used for intergroup comparisons. Spearman's rank correlation coefficient was used to analyze the correlations among non-normally distributed variables. Correlation analyses were performed using the following variables: peripheral white blood cell (WBC) count, neutrophil count, Lymphocyte count, CRP, PCT, CSF white blood cell count, CSF protein level, CSF Sugar, CSF Chlorides, and CSF Lactate dehydrogenase.

Two-tailed tests were used for all statistical analyses, and a *P*-value< 0.05 was considered to indicate a statistically significant difference.

## 3 Results

A total of 216 children with HFMD were enrolled in this study. The majority of patients were aged below 4 years. Among them, 61 cases (25.93%) were aged <1 year, 61 cases (28.24%) aged 1–2 years, 40 cases (18.52%) aged 2–3 years, and 34 cases (15.74%) aged 3–4 years. (Table 1).

**Table 1:** The age distribution trend of children with hand-foot-mouth disease.

Age (year)	Number of cases	Percentage (100%)	Cumulative percentage (100%)
<1	56	25.93	25.93
1–2	61	28.24	54.17
2–3	40	18.52	72.69
3–4	34	15.74	88.43
4–5	12	5.56	93.98
5–6	6	2.78	96.76
6–7	1	0.46	97.22
7–8	2	0.93	98.15
8–9	1	0.46	98.61
9–10	1	0.46	99.07
10–11	1	0.46	99.54
11–12	1	0.46	100.00
Total	216	100.00	100.00

Among all enrolled patients, there were 96 males (44.44%). In terms of seasonal onset, 115 cases (53.24%) developed the disease in spring, 32 cases (14.81%) in summer, 46 cases (21.30%) in autumn, and 23 cases (10.65%) in winter. Regarding residential environment, 51 cases (23.61%) resided in rural areas and 165 cases (76.39%) in urban areas. All patients were further stratified into four age groups for subsequent analysis: ≤1 month group, 1 month–1 year group, 1–3 years group, and >3 years group. Intergroup comparative analysis revealed no statistically significant differences in gender, residential environment among the four age groups. In contrast, a notable difference was identified in the season of disease onset: the ≤1 month group had a significantly higher incidence of summer onset and a markedly lower incidence of autumn onset compared with the other three age groups (Table 2).

**Table 2:** Comparison of baseline characteristics among patients of different age groups.

Variables [n(%)]	≤1 month (n = 30)	1 month–1 year old (n = 26)	1–3 years old (n = 135)	>3 years old (n = 25)	*P*
Gender					0.112
Female	14 (46.70)	20 (76.90)	73 (54.10)	13 (52.00)	
Male	16 (53.30)	6 (23.10)	62 (45.90)	12 (48.00)	
Season					0.002
Spring	18 (60.00)	17 (65.38)	66 (48.89)	14 (56.00)	
Summer	11 (36.67)	2 (7.69)	15 (11.11)	4 (16.00)	
Autumn	0 (0.00)	3 (11.54)	38 (28.15)	5 (20.00)	
Winter	1 (3.33)	4 (15.38)	16 (11.85)	2 (8.00)	
residential environment					0.687
Urban area	25 (83.33)	21 (80.77)	100 (74.07)	19 (76.00)	
Rural areas	5 (16.67)	5 (19.23)	35(25.93)	6(24.00)	

The mean duration of fever for the total cohort was 2.797 ± 2.128 days, with the shortest duration observed in the group with duration ≤1 month and the longest duration in the group>3 years old, showing statistically significant differences among groups. The median maximum body temperature for the total population was 39.00 °C (38.30, 39.40), while that for the ≤1 month group was 38.60 °C (38.00, 39.00), with a statistically significant difference in median maximum body temperature observed across all age groups.

The median hospital stay was 5.00 days (4.00, 7.00) for the total cohort, and 8.00 days (6.00, 10.00) for the ≤1 month group; the hospital stay in the ≤1 month group was significantly longer than that in the other age groups (*P* < 0.001). The group aged ≤1 month was dominated by cerebrospinal fluid (CSF) testing, with a significantly lower probability of negative results compared with other groups. By contrast, the pathogen type of HFMDin this group was similar to that in other age groups, with other types being predominant. In the ≤1 month group, the incidence of rash and pharyngeal herpes was statistically significantly lower than that in other age groups. With regard to the incidence of severe complications, the >3 years group had a markedly higher incidence of intracranial hypertension (24.00%) than the other groups (*P* < 0.001). The proportion of patients with meningeal irritation signs in the ≤1 month group reached 66.67%, which was significantly higher than that in the other groups (*P* < 0.001), and the incidence of aseptic meningitis in the ≤1 month group was 73.33%, also significantly higher compared with the other groups (*P* < 0.001). Additionally, the 1–3 years group had the highest incidence of convulsions (21.48%) among all age groups (*P* < 0.001). No statistically significant differences were observed in the other clinical manifestations across the groups (Table 3).

**Table 3:** Comparison of clinical manifestations among patients of different age groups.

Variables [(Mean ± SD)/M (Q_1_, Q_3_)/n(100%)]	≤1 month (*n* = 30)	1 month–1 year old (*n* = 26)	1–3 years old (*n* = 135)	>3 years old (*n* = 25)	*P*
Duration of fever(d)	0.46 (0.25, 1.00)	1.50 (1.00,2.25)	1.00 (1.00,2.00)	2.00 (1.0,3.00)	<0.001
Highest body temperature(℃)	38.60 (38.00,39.00)	39.00 (38.20,39.40)	39.00 (38.50,39.50)	38.70 (38.00,39.20)	0.006
Hospital stayM (d)	8.00 (6.00,10.00)	5.00 (4.00,7.00)	5.00 (4.00,6.00)	5.00 (4.00,6.00)	<0.001
Test specimen					
Throat swab	5 (16.67)	25 (96.15)	133 (98.52)	20 (80.00)	<0.001
Cerebrospinal fluid	25 (83.33)	1 (3.85)	2 (1.48)	5 (20.00)	
Types of enteroviruses					
Negative	1 (3.33)	8 (30.77)	33 (24.44)	6 (24.00)	0.014
EV71	0 (0.00)	1 (3.85)	0 (0.00)	0 (0.00)	
CV A16	0 (0.00)	1 (3.85)	9 (6.67)	0 (0.00)	
Other types	29 (96.67)	16 (61.54)	93 (68.89)	19 (76.00)	
Pharyngeal herpes	9 (30.00)	22 (84.62)	125 (92.59)	22 (88.00)	<0.001
Rash	15 (50.00)	23 (88.46)	107 (79.26)	18 (72.00)	0.005
Myoclonus	0 (0.00)	3 (11.54)	8 (5.93)	1 (4.00)	0.302
Abnormal breathing	0 (0.00)	0 (0.00)	4 (2.96)	0 (0.00)	1.000
Abnormal digestion	4 (13.33)	1 (3.85)	7 (5.19)	4 (16.00)	0.102
Dizzy giddy	0 (0.00)	0 (0.00)	1 (0.70)	1 (4.00)	0.285
Abnormal state of consciousness	3 (10.00)	1 (3.85)	12 (8.89)	0 (0.00)	0.444
Intracranial hypertension	1 (3.33)	1 (3.85)	1 (0.74)	6 (24.00)	<0.001
Dystonia	0 (0.00)	1 (3.85)	4 (2.96)	3 (12.00)	0.099
Meningeal irritation sign	20 (66.67)	0 (0.00)	1 (0.74)	3 (12.00)	<0.001
Aseptic encephalitis	3 (10.00)	1 (3.84)	5 (3.70)	2 (8.00)	0.355
Tachycardia	0 (0.00)	2 (7.69)	1 (0.74)	0 (0.00)	0.102
Aseptic meningitis	22 (73.33)	0 (0.00)	1 (0.74)	4 (16.00)	<0.001
Convulsion	0 (0.00)	1(3.84)	29(21.48)	2(8.00)	<0.001
Die	0(0.00)	0(0.00)	1(0.74)	0(0.00)	1.000

Analysis of the most abnormal values during the disease course for peripheral blood inflammatory markers revealed the following median levels in the total cohort: WBC count 10.58 × 10^9^/L, neutrophil count 6.49 × 10^9^/L, lymphocyte count 2.59 × 10^9^/L, PCT 0.18 ng/mL, and CRP 9.83 mg/L. Intergroup comparisons across the four age groups showed a statistically significant difference in peripheral blood WBC and lymphocyte counts: the ≤1 month group had the lowest WBC and lymphocyte counts, while the 1 month to 1 year group had the highest levels of both indicators (all *P* < 0.05). No statistically significant differences were observed in neutrophil count, PCT, or CRP levels among the four groups (Table 4).

**Table 4:** Analysis of differences in infection Index levels among different age groups.

Variables M (Q_1_, Q_3_)	≤1 month (*n* = 30)	1 month–1 year old (*n* = 26)	1–3 years old (*n* = 135)	>3 years old (*n* = 25)	*P*
WBC(10^9^/L)	8.69 (6.07, 11.47)	12.56 (9.83, 16.60)	11.23 (7.73, 15.27)	10.02 (7.86, 12.32)	0.005
Neutrophil(10^9^/L)	4.75 (3.19, 6.50)	7.41 (2.77, 10.59)	6.65 (3.58, 9.75)	7.87 (4.51, 9.83)	0.071
Lymphocyte(10^9^/L)	1.77 (1.50, 3.25)	4.20 (2.54, 5.75)	2.75 (1.74, 3.96)	1.74 (1.07, 2.85)	<0.001
Procalcitonin(ng/mL)	0.21 (0.11, 0.43)	0.16 (0.08, 0.45)	0.19 (0.10, 0.44)	0.12 (0.05, 0.27)	0.272
CRP(mg/L)	6.20 (2.50, 14.95)	20.05 (2.25, 42.83)	9.90 (4.17, 25.00)	5.80 (3.00, 14.90)	0.168

WBC, white blood cell; CR, C-reactive protein.

**Figure 1:**
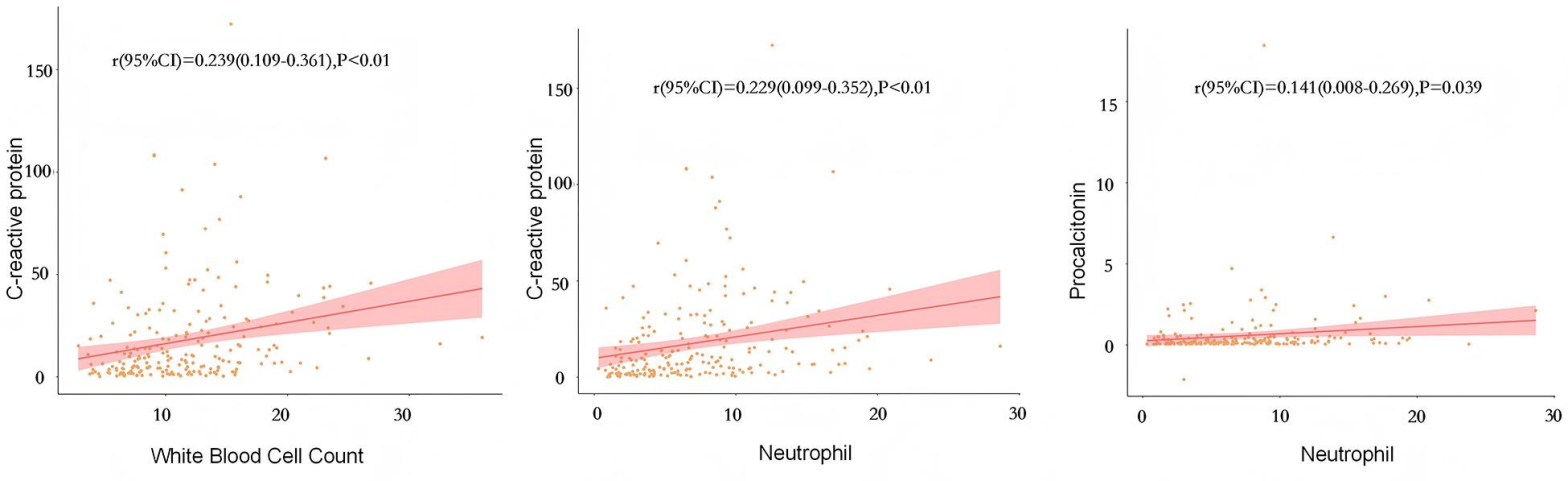
Correlation analysis of infection indicators.

Correlation analysis of peripheral blood infectious markers in all patients demonstrated significant positive correlations between WBC count and CRP, neutrophil count and CRP, as well as neutrophil count and PCT (all *P* < 0.05) (Figure 1).

Lumbar puncture and CSF examination were performed only in children with suspected neurological involvement. CSF examinations were performed in 51 of the 216 patients. The median levels of CSF and matched peripheral blood indicators in these 51 patients were as follows: CSF WBC count 3.00 × 10^9^/L, CSF protein 0.59 g/L, CSF glucose 3.10 mmol/L, CSF chloride ion 121.05 mmol/L, CSF lactate dehydrogenase (LDH) 403.00 U/L. Intergroup analysis of the 51 patients who underwent CSF testing showed notable age-stratified differences in partial CSF and peripheral blood indicators: the neonatal group had significantly higher CSF protein levels than the other groups (*P* < 0.05); CSF glucose levels were the lowest in the neonatal group and the highest in the 1–3 years group, with a statistically significant difference across groups (*P* < 0.05). For peripheral blood indicators in this subgroup, the neonatal group had the lowest WBC and neutrophil counts, while the 1 month to 1 year group had the highest WBC count (*P* = 0.007) and the >3 years group had the highest neutrophil count (*P* < 0.001). Peripheral blood lymphocyte counts were the lowest in the >3 years group and the highest in the 1 month to 1 year group, with a statistically significant intergroup difference (*P* = 0.024). No statistically significant differences were found among the four groups in CSF WBC count, CSF chloride ion concentration, CSF LDH, peripheral blood PCT, or peripheral blood CRP levels (Table 5).This negative finding should be interpreted cautiously due to small subgroup sample size.

**Table 5:** Differences in cerebrospinal fluid test results among different age groups.

Variables M (Q_1_, Q_3_)	≤1 month (*n* = 28)	1 month - 1 year old (*n* = 4)	1–3 years old (*n* = 13)	>3 years old (*n* = 6)	*P*
WBC(10^6^/L)	3.00 (1.00, 33.25)	13.50 (2.00, 27.75)	2.00 (1.00, 4.00)	42.50 (11.00, 95.00)	0.273
Protein(g/L)	0.74 (0.60, 0.95)	0.44 (0.32, 0.60)	0.20 (0.17, 0.24)	0.28 (0.23, 0.33)	<0.001
Sugar(mmol/L)	2.90 (2.65, 3.10)	3.50 (3.13, 3.80)	4.15 (3.53, 4.58)	3.30 (2.92, 3.38)	0.005
Chlorides(mmol/L)	120.55 (120.08, 121.72)	122.85 (120.82, 125.12)	121.00 (118.50, 124.08)	123.00 (122.62, 123.75)	0.056
Lactate dehydrogenase(U/L)	401.85 (329.57, 466.25)	385.00 (343.00, 496.00)	447.00 (312.00, 501.00)	344.50 (293.25, 390.50)	0.590

WBC, white blood cell.

**Figure 2:**
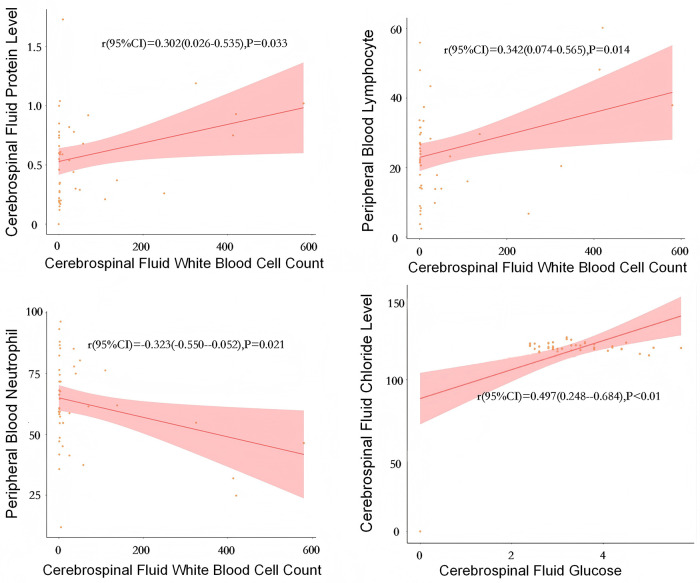
Correlation analysis between cerebrospinal fluid and infection indicators in 51 children.

Correlation analysis between CSF and peripheral blood indicators in the 51 patients identified several significant correlations: CSF WBC count was positively correlated with CSF protein level [R(95%CI) = 0.302(0.026, 0.535), *P* = 0.033] and peripheral blood lymphocyte count [R(95%CI) = 0.342(0.074, 0.565), *P* = 0.014], and negatively correlated with peripheral blood neutrophil count [R(95%CI)=−0.323 (−0.550, −0.052), *P* = 0.021]. Additionally, a significant positive correlation was observed between CSF glucose and CSF chloride ion levels [R(95%CI) = 0.497(0.248, 0.684), *P* < 0.001] (Figure 2).

The use of antibiotic was mainly observed in the neonatal group. In antiviral treatment, the neonatal group was significantly lower than the other groups; the application of glucocorticoid in the neonatal group was significantly lower than that in the other groups; while there was no significant difference in the treatment of mannitol and gamma globulin among the four groups. (Table 6).

**Table 6:** The differences in treatment situations among different age groups.

Variables, *n* (100%)	≤1 month (*n* = 30)	1 month - 1 year old (*n* = 26)	1–3 years old (*n* = 135)	>3 years old (*n* = 25)	*P*
Antibiotic	19 (63.33)	0 (0.00)	0 (0.00)	0 (0.00)	<.001
Anti Viral	2 (6.67)	17 (65.39)	55 (40.74)	11 (44.00)	<.001
Mannitol	0 (0.00)	4 (15.39)	12 (8.89)	2 (8.00)	0.163
Glucocorticoid	0 (0.00)	17 (65.39)	58 (42.96)	5 (20.00)	<.001
Gamma Globulin	2 (6.67)	1 (3.85)	5 (3.70)	0 (0.00)	0.735

## 4 Discussion

HFMD is a common infectious disease in children, whose clinical phenotypes are jointly influenced by age, EV genotyping and other factors. Based on retrospective clinical data from 216 hospitalized children with HFMD, this study systematically elucidated the spectrum of core clinical characteristics from neonates to older children through age-stratified analysis, thereby providing a critical evidence base for age-oriented precision diagnosis and treatment of HFMD.

This study confirmed a distinct age clustering pattern in HFMD incidence. Data showed that approximately 88.43% of the enrolled patients were under 4 years of age, with the 1–2 years group accounting for the highest proportion (28.24%), reflecting that infancy is a period of high susceptibility to HFMD. An analysis of 160,619 pediatric HFMD cases in the East China region from 2009 to 2015 revealed that CV-A16 and EV-A71 exhibited the highest age-specific infectivity in children aged 1 or 2 years, which is largely consistent with the age distribution observed in our study (14). This age-related susceptibility may be attributed to the expanded activity range and increased opportunities for contact transmission in this pediatric population, coupled with the decline of maternal antibodies and the immature development of their innate immune system. Notably, the neonatal group also constituted a certain proportion of cases; in addition to horizontal transmission, the potential for vertical transmission of EV in this group cannot be ignored (15, 16).

Our results demonstrated that the incidence of HFMD peaked in spring, with a secondary peak in autumn, and the seasonal peak of HFMD exhibited a significant age correlation across different age groups. The neonatal group had the lowest proportion of autumn onset and a significantly higher proportion of summer onset compared with other age groups. A key driver of the autumn incidence peak is the start of the school semester, where the high aggregation of children in kindergartens and nurseries leads to rapid viral transmission. In contrast, neonates remain in a state of home isolation, resulting in a far lower exposure risk than socially active children. The clustered summer onset in the neonatal group may be associated with the enhanced survival rate of EV on object surfaces under the high temperature and humidity of summer. Meanwhile, the immature skin and mucosal barrier of neonates increases the probability of contact with contaminated items during neonatal care, further elevating infection risk.

Regarding residential environment, the proportion of patients from urban areas was significantly higher than that from rural areas across all age groups, which is consistent with epidemiological data from other large-scale HFMD outbreaks (17–19). This discrepancy may be due to increased opportunities for contact transmission caused by high population mobility and abundant gathering activities for children in urban areas. Differences in seeking medical assistance or the severity of conditions during visits between rural and urban populations might also contribute to the disparity between the rural-urban disparities.

Age-stratified analysis revealed remarkable heterogeneity in the clinical manifestations of HFMD. Fever, the most common initial symptom, showed no statistically significant difference in duration across age groups, while the peak body temperature was significantly higher in the 1 month–1 year and 1–3 years groups than in other groups. The incidence of herpangina in the neonatal group was only 30.00%, which was significantly lower than that in the 1 month–1 year group (84.62%), 1–3 years group (92.59%) and >3 years group (88.00%). Rash, the hallmark sign of HFMD, also had a significantly lower incidence in the neonatal group compared with other age groups. This difference may be related to the immature development of oral mucosal epithelial cells in neonates and the mild inflammatory response induced by EV replication, suggesting an intrinsic difference in the pathological host response to EV infection between neonates and older children. Clinically, vigilance for HFMD without typical herpes is warranted to avoid misdiagnosis due to the absence of characteristic rash; for febrile neonates without rash, EV infection should be included in the differential diagnosis.

The age-distribution characteristics of neurological complications represent one of the core findings of this study. The >3 years group had a significantly higher incidence of intracranial hypertension (24.00%) than younger groups, and the incidence of meningeal irritation signs (12.00%) was significantly higher than that in the 1 month–1 year and 1–3 years groups but significantly lower than that in the neonatal group. The underlying mechanism by which older children are more prone to these severe complications has not been fully elucidated, but it is speculated to be associated with age-related immune responses. The more mature immune system of older children may trigger a more robust inflammatory response in response to viral infection, thereby leading to blood-brain barrier disruption and aggravated central nervous system (CNS) involvement. In contrast, the neonatal group was characterized by a high incidence of aseptic meningitis and positive EV nucleic acid in CSF, yet with a relatively benign clinical course. In addition, the 1–3 years group had the highest incidence of convulsions (21.48%), which may be related to the developmental characteristics of the nervous system and high fever sensitivity in children of this age. In terms of hospital stay, the median duration in the ≤1 month group reached 8 days, significantly longer than that in other groups (all 5 days). The prolonged hospitalization of newborns and the high rate of antibiotic treatment beyond the need for more cautious clinical observation of neonates, the prolonged hospital stay is essentially a result of balancing the risk of misdiagnosis and overtreatment: febrile neonates require simultaneous screening for bacterial sepsis, and antibiotics are typically discontinued only after negative bacterial culture results (approximately 48–72 h). Discontinuing antibiotics solely based on positive EV nucleic acid may pose a risk of missing bacterial co-infection. This conclusion provides a reference for determining the optimal hospital stay for neonates with HFMD. So Analysis of peripheral blood infectious markers showed that the 1 month to 1 year group had the highest median peripheral blood WBC count (12.56 × 10^9^/L) and lymphocyte count (4.20 × 10^9^/L), while the ≤1 month group had the lowest WBC: 8.69 × 10^9^/L; lymphocytes: 1.77 × 10^9^/L). This difference may be attributed to the active state of the immune system in children aged 1 month to 1 year, which elicits a stronger lymphocyte proliferative response to viral infection. Furthermore, this study found a significant positive correlation between peripheral blood WBC and CRP, as well as between neutrophils and procalcitonin PCT (all *P* < 0.05), suggesting that these three indicators can be used in combination to assess the degree of inflammation in children with HFMD. Especially for young children who cannot cooperate with CSF testing, this combined indicator panel can serve as an indirect basis for judging the presence of CNS infection. Age-related differences in CSF indicators have crucial diagnostic value for HFMD: the median CSF protein level in the ≤1 month group was 0.74 g/L, significantly higher than that in other groups (0.20–0.44 g/L), which is presumably due to the high permeability of the neonatal blood-brain barrier, where meningeal inflammation induced by EV infection easily leads to increased protein extravasation. In contrast, the >3 years group had the highest median WBC of CSF count (42.50 × 10^6^/L), indicating a more severe CNS inflammatory response in this group.

The neonatal group had significantly higher CSF protein levels and the lowest CSF glucose levels compared with other age groups, which is associated with the high permeability of the neonatal blood-brain barrier and the physiological characteristics of neonatal CSF. Notably, despite elevated CSF protein in the neonatal group, CSF WBC count was not significantly increased, which reconfirms that CSF cell count is not a reliable indicator for diagnosing neonatal EV meningitis, and CSF EV nucleic acid detection has irreplaceable diagnostic value. In terms of viral genotyping distribution, the proportion of non-EV71/non-CVA16 EV infections in the neonatal group was as high as 96.67%, while the detection rates of EV71 and CVA16 in the overall population were extremely low (0.46% and 4.63%, respectively). This finding has important guiding significance for the viral detection strategy of HFMD: clinical practice should expand the scope of EV genotyping detection instead of focusing solely on EV71 and CVA16. Correlation analysis of CSF indicators showed that CSF WBC count was positively correlated with CSF protein level and peripheral blood lymphocyte count, but negatively correlated with peripheral blood neutrophil count, which may imply that lymphocytes play a major role in cellular infiltration during viral meningitis. The positive correlation between CSF glucose and chloride ion levels reflects the intrinsic balance of CSF biochemical indicators.

The findings of this study have important implications for clinical practice. First, it emphasizes the necessity of age-stratified management for children with HFMD. For neonates with unexplained fever, even in the absence of typical rash or normal CSF cell count, especially in summer or with a history of exposure, CSF EV nucleic acid detection should be performed as early as possible to avoid misdiagnosis. Second, for older children (>3 years), vigilance for the risk of severe neurological complications such as intracranial hypertension and meningitis is required; enhanced disease monitoring should be implemented, and cranial imaging examinations should be performed in a timely manner when necessary. In terms of treatment, for children diagnosed with pure EV infection, especially neonates, prolonged antibiotic courses based on nonspecific inflammatory indicators (e.g., mild elevation of CRP or mild abnormality of CSF cell count) should be avoided.

It is necessary to pay attention to potential confounding factors. Firstly, treatment regimens such as antibiotics, antiviral drugs, intravenous immunoglobulin, and glucocorticoids vary among different age groups, which may interfere with the comparative analysis of hospital stay duration and complication rates. Secondly, the time span of this study is from 2022 to 2024. Annual epidemiological changes and the widespread vaccination, as well as minor adjustments in clinical diagnosis and treatment methods, may also affect the final observation results. Due to the retrospective study design, this research has not conducted a comprehensive correction for these confounding factors.

This study also has several limitations. First, this study was a single-center retrospective design without prospective sample size calculation, which may lead to potential selection bias. Second, the four age groups were highly imbalanced in sample size, and some CSF subgroups were extremely small (notably only 4 patients in the 1 month–1 year CSF subgroup), resulting in insufficient statistical power for subgroup comparisons; therefore, all negative findings should be interpreted cautiously. Third, viral genotyping was limited and did not cover all enterovirus serotypes, restricting further analysis of virus-age-manifestation associations. Although statistical methods were adjusted to accommodate small and unequal groups, our findings require validation in future multicenter, large-sample prospective cohort studies with comprehensive viral genomic and mechanistic analyses.

## 5 Conclusion

Through age-stratified analysis of 216 hospitalized children with HFMD, this study systematically elucidated significant age-specific differences in core clinical characteristics across distinct pediatric age groups. We found that the neonatal group was characterized by a benign clinical course and a high positive rate of EV nucleic acid in CSF, while the >3 years group had a markedly higher incidence of severe neurological complications, such as intracranial hypertension and aseptic meningitis. Additionally, laboratory indicators of the study population presented distinct age-specific patterns. These findings confirm that age-stratified clinical management is of crucial clinical value for children with HFMD, which can provide targeted evidence-based support for the early identification of high-risk populations, optimization of diagnosis and treatment strategies, and reduction in the unnecessary use of antibiotics in clinical practice.

## Data Availability

The datasets generated during the current study are available from the corresponding author on reasonable request.
